# Gender Differences in Mathematics Motivation: Differential Effects on Performance in Primary Education

**DOI:** 10.3389/fpsyg.2019.03050

**Published:** 2020-01-29

**Authors:** Susana Rodríguez, Bibiana Regueiro, Isabel Piñeiro, Iris Estévez, Antonio Valle

**Affiliations:** ^1^Department of Psychology, University of A Coruña, A Coruña, Spain; ^2^Department of Pedagogy and Didactics, University of Santiago de Compostela, Santiago de Compostela, Spain; ^3^Department of Pedagogy and Didactics, University of A Coruña, A Coruña, Spain

**Keywords:** gender differences, mathematics, academic motivation, academic performance, primary education

## Abstract

In addition to attempting to verify gender differences, this study aims to examine the explanatory potential of boys’ and girls’ attitudes toward mathematics on their performance. The sample comprised 897 students in the 5th and 6th years of primary education (450 boys and 447 girls). The results confirm what previous research has suggested, that girls tended to exhibit less positive attitudes about mathematics than their male classmates, in particular lower motivation, worse perception of competence, and higher rates of anxiety, although in all cases the effect sizes were small. Even though there no significant gender differences in academic performance, as expected, the explanatory power of attitudes toward mathematics was clearly more significant in boys than in girls (*R*^2^ = 0.194 and *R*^2^ = 0.103, respectively). The results of the regression analysis for each sample reinforce the well-known positive impact of perceived self-efficacy on mathematics performance and introduce the effect of achievement emotions of academic performance. Test anxiety in mathematics seems to only have a negative effect on boys’ grades, as this variable does not appear in the regression equation when explaining girls’ performance. In the light of control-value theory, we discuss the contingency of perceived competence and its involvement in anxiety and academic performance. Boys’ results could be affected by the levels of anxiety inasmuch as they tend to be confident in their abilities, motivated to stand out, and interested in mathematics. Whereas despite girls reporting high rates of anxiety, what may have a negative impact on their results might have more to do with a higher value placed on mathematics, as their perception of control may be low.

## Introduction

Spanish students’ scores in the mathematics tests in Programme for International Student Assessment (PISA) 2015 (Ministerio de Educación Cultura y Deporte [MECD], 2016) do not help us to be optimistic about the teaching and learning in this subject in our country. The score in the mathematics test from May 2015, which evaluated 37,205 of the 414,276 15-year-old students in Spain, was 486 points, significantly lower than the OECD average of 490 points. In a ranking of the 36 member countries, Spain is in 26th position, and only 7.2% of Spanish students reached high levels of achievement (5 and 6) in mathematics, which is 3.5 percentage points less than the OECD average (10.7%).

These results seem to reflect a problem that continually calls into question the effectiveness of teaching–learning processes for mathematics content. Motivational and emotional variables, which involve beliefs, emotions, and attitudes, seem to be important when it comes to fully understanding and explaining the results. In fact, everything points toward math achievement being related to variables such as perceived competence and self-efficacy (Randhawa et al., 1993; Pajares and Graham, 1999; Fast et al., 2010; Williams and Williams, 2010; Parker et al., 2014), interest (Köller et al., 2001; Lee et al., 2014; Jansen et al., 2016) and anxiety (Ashcraft, 2002; Ashcraft and Ridley, 2005; Lyons and Beilock, 2012; Ramirez et al., 2016).

In this context, when we look at the results of PISA 2015 (Ministerio de Educación Cultura y Deporte [MECD], 2016), the difference in mathematics performance between boys and girls aged 15 is an average of 8 points in OECD countries and 11 points in the European Union (EU) overall, with boys scoring higher than girls. In Spain, boys scored 16 points higher than girls, much higher than the OECD average. Various meta-analyses in the 1970s and 1980s demonstrated a slight male advantage in mathematics in secondary students (Hyde et al., 1990; Hedges and Nowell, 1995), at least in complex problem-solving tasks. However, this varies by country (Else-Quest et al., 2010; Mullis et al., 2012; Ministerio de Educación Cultura y Deporte [MECD], 2016), and more recent data suggest that this gender gap may be disappearing (Hyde et al., 2008; Hyde and Mertz, 2009; Lindberg et al., 2010).

It is an established fact that beliefs and attitudes can have a significant impact on the decision to choose a professional career related to mathematics (Colbeck et al., 2001; Ceci and Williams, 2011; Sadler et al., 2012; Kanny et al., 2014; Legewie and DiPrete, 2014; Wang and Degol, 2017). In addition, women are often underrepresented in STEM programs (science, technology, engineering, and mathematics programs), particularly in engineering, physical sciences, and computational sciences in western universities (e.g., Larivière et al., 2013; Hyde, 2014). With that in mind, it is essential to include this topic in the research agenda of educational psychology in order to thoroughly understand the interaction between motivational constructs and each group’s performance (girls and boys). In addition, research suggests that gender differences in mathematics ability are minimal during early childhood and do not consistently arise until middle to late adolescence (e.g., Lindberg et al., 2010; Robinson and Lubienski, 2011). The current study addresses the need to delve into the affective-motivational dimension of students in the final years of primary education, as girls may be losing their motivation for mathematics as they advance through this particular educational stage.

### Academic Motivation and Performance in Mathematics

Motivational research in mathematics has addressed constructs such as self-efficacy, which indicates students’ judgments of their abilities to perform specific mathematical tasks, and interest in mathematics, as positive affective experiences in activities related to mathematics. As laid out in *expectancy-value* theory, both expectations of success and subjective values related to the task directly influence performance, attainment, effort, and persistence (Wigfield and Eccles, 2000). Various studies have shown that perceived self-efficacy positively predicts academic achievement in mathematics (Randhawa et al., 1993; Pajares and Graham, 1999; Stevens et al., 2006; Fast et al., 2010; Williams and Williams, 2010; Parker et al., 2014). Similarly, students’ interest in mathematics is associated with a strong preference for mathematics content, which translates to sustained commitment over time and better performance (Köller et al., 2001; Hidi and Renninger, 2006; Lee et al., 2014; Jansen et al., 2016) in both childhood and adolescence (Lepper et al., 2005; Aunola et al., 2006; Denissen et al., 2007; Viljaranta et al., 2009).

There is also evidence that math anxiety is negatively related to performance, leading to avoidance and diminishing working memory resources needed to deal with mathematical tasks (Ashcraft, 2002; Ashcraft and Ridley, 2005; Lyons and Beilock, 2012; Ramirez et al., 2016). The nuances of the research must be understood when it comes to operationalizing the measure of math anxiety, as must the fact that the full spectrum of emotional reactions may be associated with it (Goetz and Hall, 2013; Pekrun and Linnenbrink-Garcia, 2014) and affect academic performance (Pekrun et al., 2014, 2017). For that reason, in this study, we address negative emotions associated with mathematics and math anxiety separately. In accordance with the *control-value theory* of achievement emotions (Pekrun, 2006), students who enjoy mathematics are assumed to focus their attention on the tasks, making better use of deep learning strategies and, therefore, getting better results. Students who are, for example, bored in mathematics classes pay less attention and make less use of learning strategies or use more superficial strategies, which leads to them having lower achievement (Pekrun, 2006; Pekrun et al., 2017; Putwain et al., 2018).

### Gender Differences in Mathematics Motivation

Research has confirmed gender differences, even in primary education, in mathematics self-concept, self-efficacy, and interest, suggesting that boys generally have better motivational profiles in mathematics than have girls (Eccles et al., 1993; Kurtz-Costes et al., 2008). The study of gender differences in mathematics motivation in these first educational phases is of particular interest. In this way, the most could be made of female students’ potential, improving the gender balance of participation in future STEM courses, which have been recognized as a critical filter for highly qualified and highly paid jobs.

According to previous research (Fredricks and Eccles, 2002; Jacobs et al., 2002; Preckel et al., 2008; Else-Quest et al., 2010; Frenzel et al., 2010; Guo et al., 2015; Ganley and Lubienski, 2016), girls report lower levels of individual interest and perceived mathematics competence. The most significant differences are in secondary school and university students rather than students in lower educational levels.

Demonstrating the need to explore gender differences in academic motivation, previous research has concluded that, for example, mathematics self-concept could be positively linked to achievement in boys but could even have a negative effect on girls’ achievement (Yoon et al., 1996). It has also demonstrated that the impact of interest on mathematics achievement may be slightly more important for girls than for boys (Ganley and Lubienski, 2016). On this point, although expectation-value theory does not develop a theoretical framework to address gender differences in particular, it may be used to facilitate the interpretation of the differential impact of self-beliefs and values (Wigfield et al., 1991, 1997; Eccles et al., 1993).

In terms of gender differences related to emotions provoked by mathematics, among which studies on math anxiety stand out, research indicates (albeit with small effect sizes) the existence of greater rates of anxiety in girls than boys during tasks involving mathematical reasoning (Hyde et al., 1990; Else-Quest et al., 2010). Assuming the possibility that these differences start as early as primary school (Yüksel-şahin, 2008; Griggs et al., 2013), research suggests the existence of a higher rate of math anxiety, and also more negative feelings and attitudes in boys than girls (Hyde et al., 1990; Nagy et al., 2008; Goetz and Hall, 2013; Goetz et al., 2013; Bieg et al., 2015). According to the principles of control-value theory, studies addressing the different impact suggest that positive emotions associated with mathematics could have a more pronounced effect on girls’ dedication (Pinxten et al., 2014) and that the rates of anxiety and negative emotions may not affect girls’ achievement as negatively as might have been expected (Goetz et al., 2013).

Based on these considerations, and apart from attempting to verify gender differences, the primary purpose of this study is to analyze the possible differential impact of the variables used to explore mathematical motivation on the academic achievement of boys and girls in primary education. Firstly, we hypothesize that there will be statistically significant differences in mathematical motivation between boys and girls. We expect the boys’ motivational pattern, in terms of perceived competence and intrinsic motivation for mathematics, to be more positive than the girls’ (Fredricks and Eccles, 2002; Jacobs et al., 2002; Preckel et al., 2008; Else-Quest et al., 2010; Frenzel et al., 2010; Guo et al., 2015; Ganley and Lubienski, 2016), and the girls to exhibit more negative feelings and greater anxiety toward mathematics than do the boys (Hyde et al., 1990; Nagy et al., 2008; Goetz et al., 2013; Bieg et al., 2015). We will examine the explanatory potential and differential incidence of competency beliefs and intrinsic motivation, along with negative feelings and anxiety over mathematics performance in boys and girls, assuming that perceived competence will have an impact on academic achievement in both cases (Randhawa et al., 1993; Pajares and Graham, 1999; Stevens et al., 2006; Fast et al., 2010; Williams and Williams, 2010; Parker et al., 2014). We will also explore the possibility that the impact of intrinsic motivation on performance will be more significant when it comes to explaining girls’ achievement rather than boys’ (e.g., Yoon et al., 1996; Ganley and Lubienski, 2016). Finally, we will also look at whether anxiety is more important when explaining boys,’ compared with girls,’ mathematics achievement (e.g., Goetz et al., 2013).

## Materials and Methods

### Participants

The sample was composed of 897 students from 13 public primary schools in the Spanish province of A Coruña. Half (50.2%) were boys and half (49.8%) were girls. They were aged between 9 and 13 years (*M* = 10.77; *SD* = 0.69). Out of the total, 437 were in the 5th year of primary education (223 boys and 213 girls) and 460 were in the 6th year of primary education (227 boys and 233 girls).

### Instruments

To measure students’ attitudes toward mathematics, we used the IAM (*Inventario de Actitudes hacia las Matemáticas/Inventory of Attitudes Toward Mathematics*). This instrument is the result of expanding the *Fennema–Sherman Mathematics Attitude Scale* (FSS) by Fennema and Sherman (1976). It is an extended version of the scale, with some modifications, adapted into Spanish and including new dimensions aimed at measuring more accurately students’ attitudes and motivation for mathematics (Silva, 2005; González-Pienda et al., 2012; Cueli et al., 2014). In this study, we used the following IAM dimensions:

• *Intrinsic motivation toward mathematics* (four items; *α* = 0.72): this evaluates motivation toward learning and understanding mathematics content for the pleasure and personal satisfaction that comes from working with this type of content (example item: “I find mathematics enjoyable and stimulating”).
• *Perceived competence in mathematics* (four items; *α* = 0.75): this evaluates the level of confidence in oneself for learning and getting good mathematics results (example item: “I think I can do even more difficult math tasks”).
• *Negative feelings caused by mathematics* (three items; *α* = 0.71): this evaluates the presence and intensity of sadness and unease caused by studying, homework, or attending math classes (example item: “In math class I am sad and unhappy”).
• *Math anxiety* (three items; *α* = 0.77): this evaluates the level of students’ fear and nervousness with math tests and tasks (example item: “I feel uncomfortable and nervous about mathematics”).

The items in each dimension were in a Likert-type format with five response options from 1 (completely false) to 5 (completely true).

The evaluation of *academic performance in mathematics* was obtained via the final school grades that the participating students received in this subject. The following grades were used: 1 = poor, 2 = satisfactory, 3 = good, 4 = very good, and 5 = outstanding.

### Procedure

The data were collected during school hours by personnel external to the school with the prior consent of the school management and the students’ teachers. Prior to participating in the study, the teachers, students, and parents (depending on school regulations) were informed of the study content and procedure. Before data collection, which was done on a single occasion, the participants were reminded of the importance of answering the various questions honestly.

Data about the target variables were collected in accordance with the recommendations of the ethical standards established in the Research and Teaching Ethics Committee of the University of A Coruña and the Declaration of Helsinki. Confidentiality of data was ensured, and participation was voluntarily such that withdrawal from the study was possible at any time.

### Data Analysis

In addition to descriptive and correlational analyses, analysis of variance (ANOVA) was also used to examine gender differences in mathematics motivation. A stepwise linear regression analysis was performed to examine the predictive capacity of the motivational variables on achievement in mathematics between boys and girls. In both cases, the predictor variables were *intrinsic motivation*, *perceived competence*, *negative feelings*, and *math anxiety*, with the criterion variable being the students’ final academic grade in mathematics. Firstly, the analysis aimed to understand the contribution of each of the variables added to the regression equation when it came to predicting mathematics performance. In addition, we wanted to ascertain the weight and specific significance of the predictor variables in each sample. Effect sizes were calculated according to the criteria in Cohen (1988) classic work: *d* < *0.20* = non-significant effect; *d* ≥ *0.20* and *d* < *0.50* = small effect; *d* ≥ *0.50* and *d* < *0.80* = moderate effect; and *d* ≥ *0.80* = large effect.

## Results

The analysis of results first looks at determining whether there are significant differences in *intrinsic motivation*, *perceived competence*, *negative feelings*, and *math anxiety* by *gender*. Following that, we examine the contribution of this set of affective-motivational variables on students’ academic performance.

Table 1 shows the correlation coefficients as well as descriptive statistics of central tendency, distribution, and dispersion for the variables in this study. Considering the correlations, academic achievement in mathematics had a positive, statistically significant relationship to *intrinsic motivation* toward mathematics (*r* = *0.19*, *p* < 0.01) and *perceived competence* (*r* = *0.28*, *p* < 0.01). *Anxiety* and *negative feelings* toward mathematics had a negative association with performance (*r* = −0.29, *p* < 0.01, and *r* = −0.33, *p* < 0.01, respectively).

**TABLE 1:** Means, standard deviations, asymmetry, kurtosis, and correlation matrix.

	1	2	3	4	5
1. Perceived competence	–				
2. Intrinsic motivation	0.68^a^	–			
3. Negative feelings	−0.43^a^	−0.50^a^	–		
4. Anxiety	−0.49^a^	−0.44^a^	0.47^a^	–	
5. Academic performance	0.28^a^	0.19^a^	−0.33^a^	−0.29^a^	–
*M*	4.04	3.71	1.77	2.10	3.41
SD	0.75	0.86	0.87	1.07	1.27
Asymmetry	–0.89	–0.50	1.27	0.93	–0.43
Kurtosis	0.79	–0.18	1.44	0.10	–0.91

*^*a*^*p* < 0.01.*

The relationship between perceived competence and both negative emotions and math anxiety was negative and significant (*r* = −0.43, *p* < 0.01, and *r* = −0.49, *p* < 0.01, respectively). Similarly, negative feelings toward mathematics and anxiety were both significantly, negatively correlated with intrinsic motivation toward mathematics (*r* = −0.50, *p* < 0.01, and *r* = −0.49, *p* < 0.01, respectively). As one might expect, there was a significant, positive association between negative feelings toward mathematics and math anxiety (*r* = 0.47, *p* < 0.01).

### Differences in Mathematics Motivation Between Boys and Girls

We found statistically significant differences in mathematics motivation between boys and girls in primary education, with a moderate effect size, even though the differences in mathematics performance were not significant [*F*_(__1_,_895__)_ = 1.174, *p* = 0.279]. Girls reported lower levels of *self-efficacy* [*F*_(__1_,_985__)_ = 11.227; *p* < 0.01; *η*_*p*_^2^ = 0.012; *d* = 0.71] and *intrinsic motivation* [*F*_(__1_,_895__)_ = 6.522; *p* < 0.05; *η*_*p*_^2^ = 0.007; *d* = 0.61] for mathematics than did boys. On the other hand, whereas both boys and girls had similar levels of *negative feelings* toward mathematics [*F*_(__1_,_895__)_ = 1.272, *p* = 0.260], the girls exhibited higher rates of *math anxiety* than did their male classmates [*F*_(__1_,_895__)_ = 11.018; *p* < 0.01; *η*_*p*_^2^ = 0.012; *d* = 0.70].

### The Impact of Motivation on Mathematics Performance in Girls and Boys

We performed two identical stepwise regression analyses, one for the sample of boys (*n* = 450; *M*_age_ = 10.79; *SD* = 0.70) and one for the girls (*n* = 447; *M*_age_ = 10.75; *SD* = 0.68), with the aim of determining the predictive value of the motivational variables on mathematics performance in each sample.

As Table 2 shows, in the boys’ group, both negative feelings and anxiety associated with mathematics, along with perceived competence in the subject, contributed significantly to the prediction of academic performance [*F*_(__3_,_446__)_ = 36.914; *p* < 0.001], explaining almost 20% of the variance (*R*^2^ = 0.194) in the criterion variable.

**TABLE 2:** Explained variance (*R*^2^), change in *R*^2^ (Δ*R*^2^), regression coefficients (*β*), and associated statistical significance [*t*(*p* <)] in the prediction of boys’ and girls’ mathematics performance.

		*R*^2^_adjust_	Δ*R*^2^	*β*	*t*(*p* <)
**Boys**					
Model 1		0.131	0.133		
	*Negative feelings*			–0.365	−8.299***
Model 2		0.176	0.046		
	*Negative feelings*			–0.253	−5.228***
	*Anxiety*			–0.242	−5.011***
Model 3		0.194	0.020		
	*Negative feelings*			–0.207	−4.156***
	*Anxiety*			–0.192	−3.819***
	*Competence*			0.162	3.301***
**Girls**					
Model 1		0.080	0.082		
	*Negative feelings*			–0.287	−6.322***
Model 2		0.093	0.015		
	*Negative feelings*			–0.228	−4.525***
	*Competence*			0.134	2.670***
Model 3		0.103	0.012		
	*Negative feelings*			–0.267	−5.087***
	*Competence*			0.225	3.625***
	*Intrinsic Motivation*			–0.159	−2.667**

**p < 0.01 and ***p < 0.001.

In the girls’ group, intrinsic motivation, negative feelings toward mathematics, and perceived competence in the subject were also predictor variables of mathematics performance [*F*_(__3_,_443__)_ = 18.093; *p* < 0.001], although in this case the percentage of explained variance was half of that in the boys’ sample (*R*^2^ = 0.103).

The results of the analyses confirm the explanatory potential of both *negative feelings* toward mathematics (β = −0.207; *t* = −4.156; *p* < 0.001 for the boys, and β = −0.267; *t* = −5.087; *p* < 0.001 for the girls) and *perceived competence* (β = *0.162*; *t* = 3.301; *p* < 0.001 for the boys, and β = 0.225; *t* = 3.625; *p* < 0.001 for the girls) in mathematics performance (see Table 2). In both samples, negative feelings were associated with poor mathematics performance, whereas the positive regression coefficient for perceived competence in the subject indicates, as previous research has noted, the positive link between these types of beliefs and performance.

The results also indicate some differential aspects that should be underscored. Firstly, the contribution of math anxiety was only an explanatory factor for the boys (β = −0.192; *t* = −3.819; *p* < 0.001), whereas intrinsic motivation appeared as a significant variable in the explanation of girls’ performance (β = −0.159; *t* = −2.667; *p* < 0.05). The negative regression coefficient of this variable indicates that, for girls, commitment to mathematics for intrinsic reasons may negatively affect their performance in this subject. We discuss this result in light of Pekrun’s (2006) control-value theory.

## Discussion and Conclusion

In line with previous research (Hyde et al., 1990; Else-Quest et al., 2010; Goetz et al., 2013; Bieg et al., 2015), we confirm that girls in the 5th and 6th years of primary education report higher levels of math anxiety than do boys, in this case, with moderate effect sizes. However, these gender differences in math anxiety may not be transferable to other negative feelings or emotions such as sadness or boredom, as previous studies have suggested (Hyde et al., 1990; Nagy et al., 2008; Goetz and Hall, 2013; Goetz et al., 2013; Bieg et al., 2015). Our results also replicate prior studies, showing that boys report greater intrinsic motivation and perceived competence for mathematics than do girls (Fredricks and Eccles, 2002; Jacobs et al., 2002; Preckel et al., 2008; Else-Quest et al., 2010; Frenzel et al., 2010; Louis and Mistele, 2012; Guo et al., 2015; Ganley and Lubienski, 2016), even though their performance in mathematics is similar (Skaalvik and Skaalvik, 2004; Else-Quest et al., 2010; Lindberg et al., 2010).

In general terms, as expected, our results confirm the well-known positive impact of perceived self-efficacy on mathematics performance at school (Zimmerman, 1995; Pajares and Graham, 1999; Valentine et al., 2004; Fast et al., 2010; Williams and Williams, 2010; Parker et al., 2014), found in both standardized tests and, as in our case, the grades awarded in class (Randhawa et al., 1993; Fast et al., 2010; Grigg et al., 2018).

In addition, as various perspectives have suggested, our results highlight the importance and influence of students’ emotions on their learning and performance. The explanatory power of negative emotions associated with mathematics has been shown to be even more important than the oft-quoted math anxiety when it comes to explaining performance in this subject. Research on emotions in mathematics has repeatedly focused on math anxiety, very probably ignoring a good number of different emotions such as sadness and boredom, which as “hot cognition,” may mark the path of learning and success. Even though studies indicate that the relationships between emotions and performance generally tend to have small or moderate effect sizes, it is possible that academic emotions end up having a significant cumulative long-term impact on performance. For this reason, we should not lose sight of their role in health, subjective well-being, choice and continuation of study, or in lifelong learning (Pekrun, 2006).

The differences in percentages of variance in academic performance explained by the motivational variables that we examined in boys and girls indicate the need to encourage research into gender differences in academic emotions and motivations in general and in mathematics in particular. Whereas negative feelings, anxiety, and perceived competence together explain almost 20% of the variance in boys’ performance in mathematics, the explanatory power of negative feelings, competence, and intrinsic motivation in girls is practically half of that. Producing a body of knowledge that would let us characterize differential motivational profiles, in contexts of achievement, would allow better adjustment of affective-motivational interventions. The gender gap in cognitive abilities could be reduced in the long term with the promotion of specific educational experiences in these first stages of formal education (Ganley and Lubienski, 2016).

In this study, the performance of the boys, who were more confident in their abilities, more motivated, and more interested in mathematics, was seen to diminish in relation to the appearance of negative feelings about mathematics, and there were high rates of anxiety. Nevertheless, and in line with observations by Goetz et al. (2013), our results seem to demonstrate that although girls reported higher levels of anxiety, their performance in mathematics tasks and tests was not affected as negatively as might have been expected. The state-trait discrepancy that research recognizes in these and other academic emotions (e.g., Porter et al., 2000; Frenzel et al., 2007; Bieg et al., 2013, 2014) suggests that gender differences found in math anxiety may not be reflected in day-to-day school learning processes. Emotional valuations may be strongly influenced by subjective beliefs such as expectations or attributional tendencies. The commonly accepted idea that in mathematics girls believe themselves to be less competent may be behind an erroneous anticipatory evaluation of their math anxiety (e.g., Skaalvik and Skaalvik, 2004; Ganley and Lubienski, 2016). These girls’ beliefs about their competence in mathematics, together with gender stereotypes (Wigfield and Eccles, 2000; Pekrun, 2006; Thoman et al., 2013; Bieg et al., 2015), contribute to an emotional evaluation that differs from the emotions really associated with the specific tasks or situations. This is why despite girls exhibiting higher levels of anxiety about mathematics than do boys, this variable is not an explanatory factor in girls’ performance.

With respect to the premises of the control-value theory, negative feelings such as sadness, unhappiness, and despair associated with mathematics tasks are surely the result of a pattern of low control with high value placed on success (Frenzel et al., 2007; Bieg et al., 2015). The inclusion of intrinsic motivation as a negative predictor of girls’ mathematics performance must be understood in this context, characterized by low perceived competence for the subject. Just as Pekrun’s (2006) theory determines, if negative emotions are the result of a pattern of low control together with high value placed on success, involvement for intrinsic reasons, indicating high value placed on mathematics, could end up decreasing girls’ performance inasmuch as it could contribute to negative feelings in this area.

## Educational Implications

In terms of the potential of emotions associated with the classroom, it is worth suggesting longitudinal, experimental, and interventional research to examine the assumed causal relationships between discrete emotions, both negative (as we have done in this study) and positive (enjoyment, hope, and gratitude among others), and performance. Apart from incorporating knowledge about moderating and mediating variables of academic emotions and their background into the teacher training syllabus, teachers should also consider the impact of educational styles on their students’ academic emotions, and their role in encouraging positive emotions and reducing negative emotions associated with the classroom.

In terms of our results, and in line with Ganley and Lubienski (2016), mathematics interventions for girls should start early and deal specifically with perceptions of confidence and control. On the other hand, management of emotions, particularly anxiety, could be extremely important for mathematics interventions for boys. The identification and development of instruction strategies and intervention plans to improve the affective-emotional experiences associated with the learning process should be on the educational and research agenda.

## Limitations

Apart from the inherent limitations of the research design, we must recognize the use of a self-report to measure emotions and the fact that boys and girls surely differ in their abilities and dispositions when reporting their emotions (Bryant et al., 1996). Information about emotions may be especially vulnerable to social desirability or to stereotyping in a context such as mathematics, which is perceived as a male domain.

## Data Availability Statement

The datasets generated for this study are available on request to the corresponding author.

## Ethics Statement

The studies involving human participants were reviewed and approved by Ethics Committee of University of A Coruña. Written informed consent to participate in this study was provided by the participants’ legal guardian/next of kin.

## Author Contributions

SR and IE contributed to collect the data, data analysis, and writing the manuscript. BR and AV contributed to data analysis and writing the manuscript. IP wrote the manuscript.

## Conflict of Interest

The authors declare that the research was conducted in the absence of any commercial or financial relationships that could be construed as a potential conflict of interest.
